# Simple fluorometric-based assay of antibiotic effectiveness for *Acinetobacter baumannii* biofilms

**DOI:** 10.1038/s41598-019-42353-0

**Published:** 2019-04-19

**Authors:** Dhammika Leshan Wannigama, Cameron Hurst, Lachlan Pearson, Thammakorn Saethang, Uthaibhorn Singkham-in, Sirirat Luk-in, Robin James Storer, Tanittha Chatsuwan

**Affiliations:** 1 Department of Microbiology, Faculty of Medicine, Chulalongkorn University, King Chulalongkorn Memorial Hospital, Bangkok, Thailand; 2 Medical School, Faculty of Health and Medical Sciences, The University of Western Australia, Nedlands, Western Australia Australia; 3 Center of Excellence in Biostatistics, Faculty of Medicine, Chulalongkorn University, Bangkok, Thailand; 4 Department of Statistics, QIMR Berghofer Medical Research Institute, Brisbane, Queensland Australia; 5 Systems Biology Center, Research Affairs, Faculty of Medicine, Chulalongkorn University, Bangkok, Thailand; 6 Snowy River Vet Clinic and Veterinary Hospital, Orbost, Victoria, Australia; 7 Interdisciplinary Program of Medical Microbiology, Graduate School, Department of Microbiology, Faculty of Medicine, Chulalongkorn University, Bangkok, Thailand; 8 Office of Research Affairs, Faculty of Medicine, Chulalongkorn University, Bangkok, Thailand; 9 Antimicrobial Resistance and Stewardship Research Unit, Faculty of Medicine, Chulalongkorn University, Bangkok, Thailand; 10 Department of Clinical Microbiology and Applied Technology, Faculty of Medical Technology, Mahidol University, Bangkok, Thailand; 11 Department of Computer Science, Faculty of Science, Kasetsart University, Bangkok, Thailand

**Keywords:** Bacterial infection, Infection, Translational research

## Abstract

Despite strengthened antimicrobial therapy, biofilm infections of *Acinetobacter baumannii* are associated with poor prognosis and limited therapeutic options. Assessing antibiotics on planktonic bacteria can result in failure against biofilm infections. Currently, antibiotics to treat biofilm infections are administered empirically, usually without considering the susceptibility of the biofilm objectively before beginning treatment. For effective therapy to resolve biofilm infections it is essential to assess the efficacy of commonly used antibiotics against biofilms. Here, we offer a robust and simple assay to assess the efficacy of antibiotics against biofilms. In the present work, we carefully optimized the incubation time, detection range, and fluorescence reading mode for resazurin-based viability staining of biofilms in 96-well-plates and determined minimal biofilm eradication concentrations (MBECs) for *A. baumannii* isolates from patients with chronic infection. By applying this assay, we demonstrated that antibiotic response patterns varied uniquely within the biofilm formation of various clinical samples. MBEC-50 and 75 have significant discriminatory power over minimum inhibitory concentrations for planktonic suspensions to differentiate the overall efficiency of an antibiotic to eradicate a biofilm. The present assay is an ideal platform on which to assess the efficacy of antibiotics against biofilms *in vitro* to pave the way for more effective therapy.

## Introduction

Every medical procedure that depends on antibiotics to fight infections can become compromised by antibiotic resistance. Bacteria have acquired increasing resistance to antibiotics since their introduction and this causes extensive illness and deaths worldwide. Among the bacteria that are alarmingly prevalent are multi-drug resistant *Acinetobacter baumannii*, which cause some 60% of hospital-acquired or nosocomial infections^1^. These bacteria have become prevalent in communities, causing ventilator associated pneumonia, blood stream and a variety of skin and tissue infections, in both healthy and immune-compromised individuals^1,2^. Indeed, the majority are chronic biofilm-associated infections that are highly resistant to antibiotic therapy, with 40–60% mortality rates^1,2^.

The biofilm structure makes it difficult for antibiotics to kill the bacteria that form biofilms, and subsequent infection can persist for up to weeks or months, and develop even greater resilience against antibiotics and spread to other organs^2–4^. The biofilms can be impenetrable to antibiotics and immune cells, and bacteria in the deeper portions of the biofilms are in a state of slow growth, which acts as a structural and physiological barrier against antibacterial agents^3,5^. The biofilm phenomenon is also responsible for producing various virulence factors that invade host immune systems to mount episodes of acute overexuberant inflammatory response^6^.

Predisposing factors for antibiotic treatment failure are numerous, ranging from biofilm recalcitrance towards treatment and lack of appropriate antibiotic selection tests^2^. The better selection of antibiotics for biofilm infection has long been elusive^7^. The type and reservoir of resistance determinants are likely to enhance biofilm formation in multi-drug resistant (MDR) *A. baumannii* isolates from patients with chronic infections^7,8^. Numerous studies performed on clinical isolates associated with nosocomial infections showed significant correlation between biofilm formation and resistance to ampicillin-sulbactam, imipenem, ceftazidime, ciprofloxacin, amikacin, and piperacillin^8–10^. *A. baumannii* biofilm strains associated with respiratory tract infections, catheter-related infections, and blood stream infections, have a higher resistance to gentamicin, minocycline, ciprofloxacin, ceftazidime, cefotaxime, imipenem, and meropenem, and reduced susceptibility to colistin^7,8,10,11^. Routine clinical selection of antibiotics is based on minimum inhibitory concentrations (MICs) for planktonic bacteria, rather than inhibition of bacteria in biofilm growth states^2,12,13^. Therefore, rapid and accurate treatment is often difficult in routine clinical practice because pathophysiological biofilm conditions are not accurately represented in MIC testing procedures.

Antibiotic regimens based on biofilm susceptibility testing highlight the remarkable improvement in clinical outcomes compared with those based on standard MIC test results, and allow physicians to identify more rapidly the appropriate antibiotic for patients with chronic biofilm infections^14–17^. It is well recognized that, a simple, rapid antimicrobial susceptibility test for biofilms is crucial for better clinical decision making to control chronic biofilm infections with appropriate antibiotic therapy^12,17,18^.

Currently, the most common method to quantify susceptibility of biofilms to antibiotics involves conventional plating and requires manual detachment of biofilms and their dispersal^19^. However, to our knowledge, there is currently no definitive, standardized, rapid method to discriminate the efficacy of antibiotics between biofilm and non-biofilm bacteria. Various research groups have developed various methods to characterize bacterial biofilm antibiotic susceptibility *in vitro*^17^. Some methods involve staining (e.g., with Crystal Violet (C.I. 42555), Syto9, or propidium iodide) to determine cell viability in biofilms via spectrophotometric analysis or using confocal laser scanning microscopy^20–22^. Other methods require specific equipment (e.g., the Calgary biofilm device and biofilm ring test)^17,20,22–24^ to characterize the minimal biofilm eradication concentration (MBEC) for various antibiotics. These methods are not so simple for routine clinical use, and may require staff with considerable expertise, complex laboratory procedures, and expensive instruments, and may result in non-specific staining of the biofilm matrix rather than viable cells. Only a very limited number of equivalent biofilms can be produced at the same time and even so with poor reproducibility^19^. Only limited clinical samples and statistical attributes have been used to claim the integrity of these methods.

Here we developed a simple fluorometric-based assay that rapidly quantifies metabolically active bacterial cells in *A. baumannii* biofilm using PrestoBlue, a resazurin (7-hydroxy-3*H*-phenoxazin-3-one-10-oxide)-based viability indicator. We rendered this simple approach into a standard reliable test by carefully combining relevant statistical attributes with a diverse variety of clinical isolates to provide an accurate and precise quantitative analysis of MIC and MBEC for various clinical sample types and antibiotics. The present assay represents a potentially definitive way of predicting how bacteria within biofilm will respond to antibiotic treatment.

## Results

### Fluorescent signals from planktonic cells are sturdier than those from cells in biofilm

**Figure 1:**
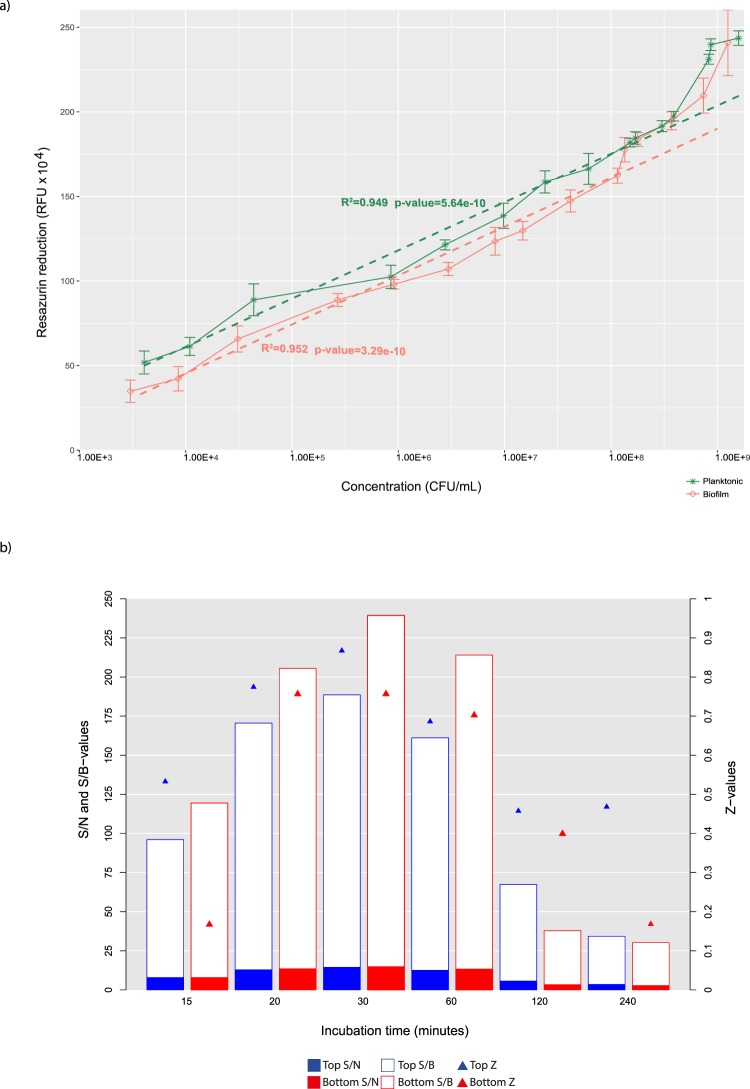
(**a**) Relationship between PrestoBlue reduction (in relative fluorescence units, RFU) and bacterial concentration (in colony forming units or CFU/mL) measured for planktonic bacteria and biofilms. (**b**) Robustness of the incubation time with PrestoBlue on the antimicrobial susceptibility assay performance, as measured by signal window coefficient, *Z*′-factor; signal-to-noise (S/N), and signal-to-background (S/B) ratios.

The amount of fluorescent resorufin produced was linearly proportional to the viable bacterial cell concentrations in both planktonic and biofilm growth conditions (Fig. 1a). A linear range was observed between 10^4^–10^8^ colony forming units (CFU) per biofilm (R^2^ = 0.952), while significant fluorescent signal was detected when bacteria concentrations (planktonic and biofilm) were <10^4^ CFU (Fig. 1a). The planktonic cells showed a stronger fluorescent signal than those within the biofilm (*p* < 0.005) (*p* = 3.98E–7).

### Incubation time and fluorescence reading mode are important to optimise fluorescent signals

The minimum incubation period required to generate a fluorescent signal adequately above background was within the range 20–30 min (Fig. 1b). In the fluorescence reading mode from above the 96-well microtitre plate (top mode), 30 min was the shortest time providing good results with a high signal window coefficient (*Z* > 0.8). In the top reading mode, a 20 min incubation was sufficient to generate adequate sensitivity (*Z* > 0.7, higher S/B and S/N). In the reading mode from the underside of the plate (bottom mode), 30 min was the shortest time providing good results with a high signal window coefficient (*Z* > 0.8). Changes in the quality of fluorescent signals (lower *Z* and S/B) between modes of reading after one hour incubation suggest interference at incubation times longer than 30 min (Fig. 1b).

### The standard colony count correlated to fluorescent signals of PrestoBlue

Linear correlation (*p* < 0.005, linear modelling analysis) between average fluorescence intensity of PrestoBlue and the CFU counts in biofilms were observed in a susceptibility test (Supplementary Fig. 1). We also found that some cells were viable at high concentrations of antibiotics and emit detectable fluorescence signals (confirmed by CFU counts) (Supplementary Fig. 2) without interference from background noise.

### Differential responses to antibiotics by planktonic bacteria and those in biofilms are typically not a result of inherited resistance

**Figure 2:**
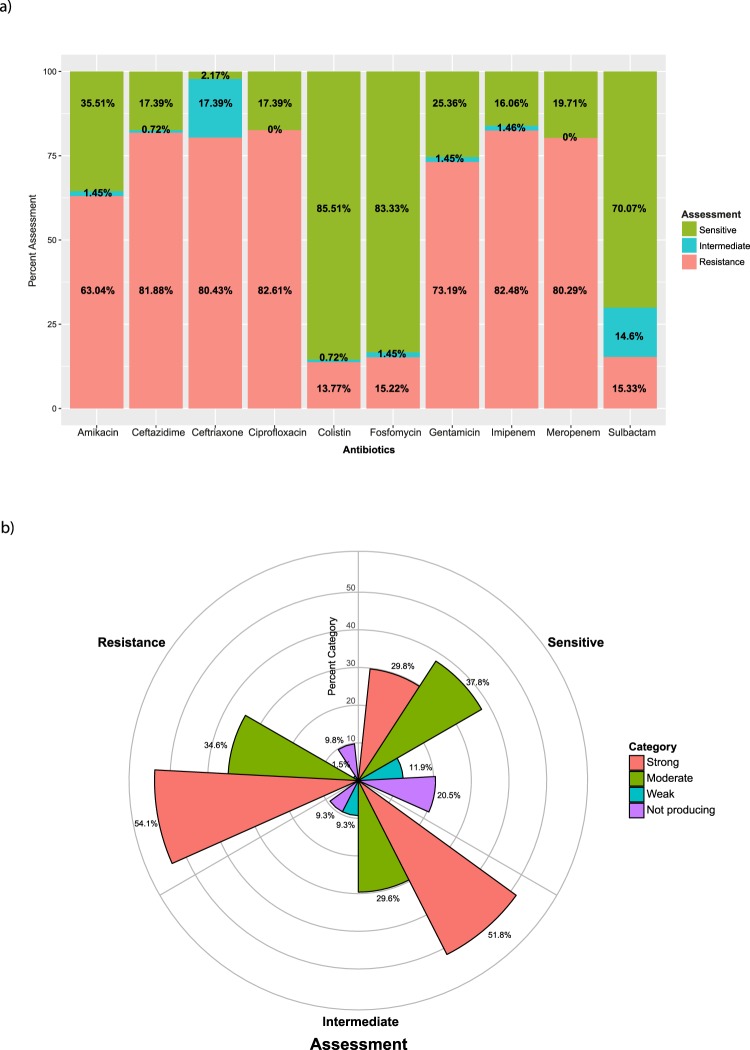
(**a**) Antibiotic susceptibility of clinical isolates of *A. baumannii* to seven antibiotics. (**b**) Distribution of the resistance among various biofilm production capacities as a percentage.

The prevalence of antibiotic resistance among the 138 resistant isolates included in the study is shown in Fig. 2a. More than 50% of the isolates showed high resistance to amikacin, ceftazidime, ceftriaxone, ciprofloxacin, gentamicin, imipenem, and meropenem, while other isolates displayed considerable intermediate susceptibility to colistin, fosfomycin, and sulbactam (Fig. 2a).

We classified 119 isolates as biofilm positive and 62 isolates as strong biofilm producers. However, within biofilm-positive isolates there was much heterogeneity in antibiotic susceptibility (Fig. 2b). We observed significant association between strong and moderate levels of biofilm production and antibiotic resistance (*p* < 0.001). Strong biofilms were dominant in intermediate and resistant isolates (*p* < 0.001). By contrast, there was a significant association between antibiotic sensitivity and isolates forming moderate biofilms (*p* < 0.001).

### Rationale for the anti-biofilm method for detecting differences in antibiotic susceptibility levels

**Figure 3:**
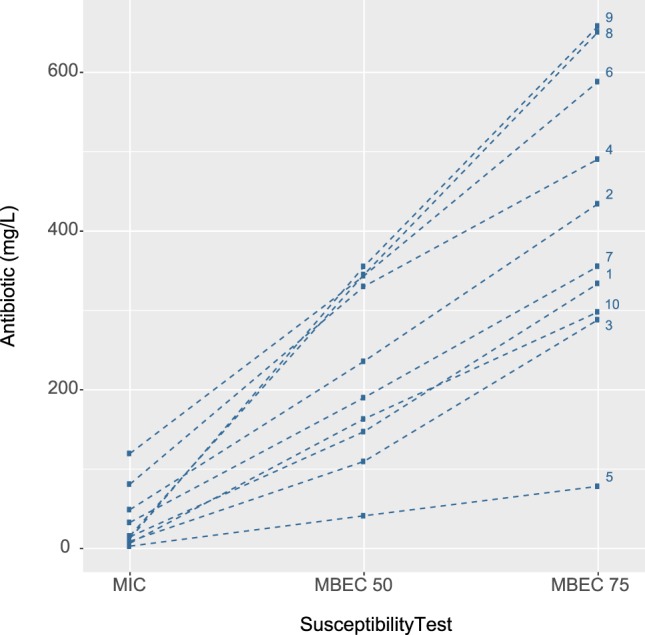
Relationship between susceptibility of *A. baumannii* clinical isolates and ten antibiotics (1, gentamicin; 2, amikacin; 3, ciprofloxacin; 4, ceftriaxone; 5, colistin; 6, fosfomycin; 7, ceftazidime; 8, imipenem; 9, meropenem; 10, sulbactam).

A significant association (*Z*^2^_LRT_ = 347.21, 18 df, *p* < 0.001) between the antibiotics and type of susceptibility test were confirmed by linear mixed modelling. Figure 3 shows the strong levels of discriminatory power between each test type was modified by antibiotics. The pattern of MBEC75 > MBEC50 > MIC to tested antibiotics is prevalent in all isolates (Fig. 3). In other instances, e.g., for colistin, the levels of discrimination between MIC and MBEC are much less prominent than for other antibiotics. Whereas for ceftriaxone, fosfomycin, imipenem, and meropenem the difference between MIC and MBEC50 is more pronounced. The MIC test has a relative paucity to differentiate antibiotic susceptibility in biofilms (Fig. 3) because the overlapping set of effective antibiotic concentrations may have constrained the possible basis for selection of appropriate antibiotics (Fig. 3). Dose–response curves for all antibiotics tested clearly demonstrated the magnitude of differences between planktonic and biofilm bacteria, emphasizing the MBEC75 > MBEC50 > MIC relationship (Supplementary Fig. 4).

### The anti-biofilm test finds a correlation between the level of biofilm formation and antibiotic responses

**Figure 4:**
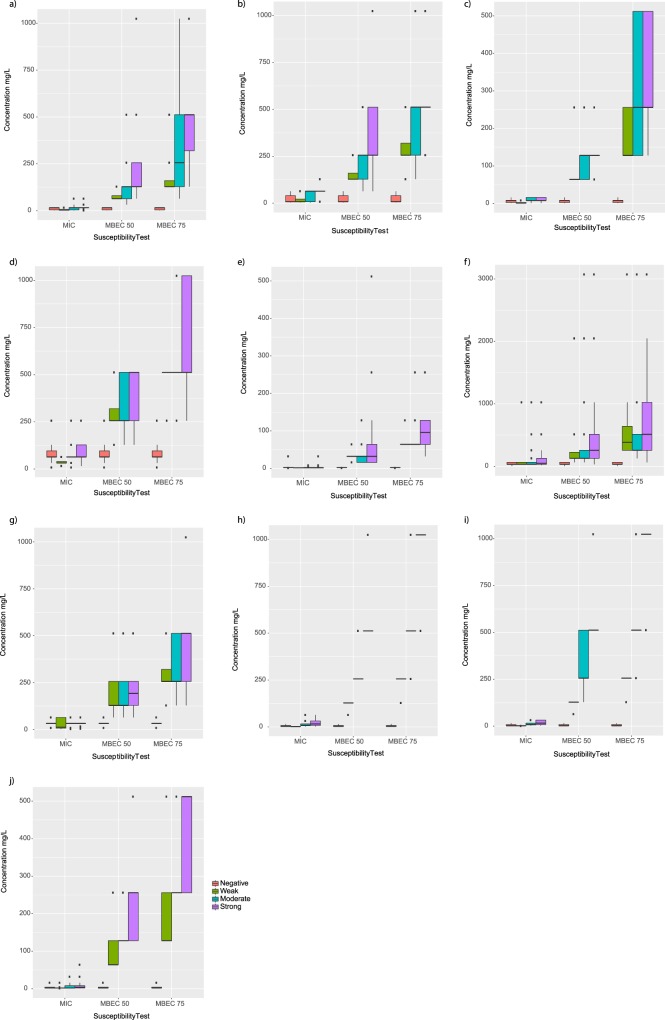
Association between the level of biofilm formation (negative, weak, moderate, or strong) and susceptibility of *A. baumannii* clinical isolates test results for ten antibiotics. (**a**) gentamicin, (**b**) amikacin, (**c**) ciprofloxacin, (**d**) ceftriaxone, (**e**) colistin, (**f**) fosfomycin, (**g**) ceftazidime, (**h**) imipenem, (**i**) meropenem, and (**j**) sulbactam.

We conducted a comparative analysis of biofilm forming capacities of each isolate and three types of susceptibility tests for each antibiotic tested (Fig. 4). Of note, all isolates showed MBEC susceptibility values were significantly modified by biofilm formation with similar direction for strong and moderate biofilms (*p* < 0.001) (Fig. 4). MIC testing was unable to discern any relevant differences in association with weak, moderate, or strong biofilms. For eight antibiotics (gentamicin, amikacin, ciprofloxacin, ceftriaxone, fosfomycin, imipenem, meropenem, and sulbactam) results showed an obvious increase (MBEC75 > MBEC50 > MIC) in effective concentration to eliminate different biofilm forming capacity (Fig. 4). Notably, isolates forming a strong and moderate biofilm had a pronounced difference in sensitivity to ciprofloxacin, ceftriaxone, and sulbactam in the MBEC75 test. This tendency differed, particularly for colistin, but also for ceftazidime and fosfomycin, which displayed less dissimilarity in antibiotic sensitivity between planktonic and biofilm states than the other antibiotics.

### Anti-biofilm tests reveal different susceptibility levels in clinical isolates exposed to the same antibiotic

**Figure 5:**
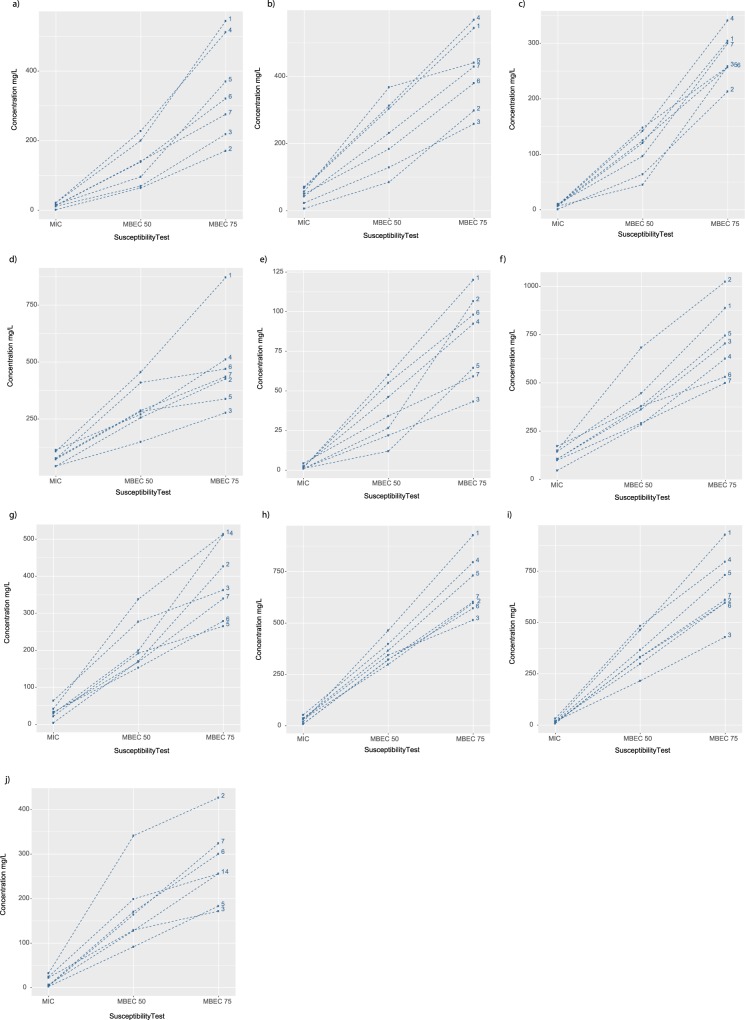
Association between the type of *A. baumannii* clinical isolate sample (1, urine; 2, nasal swabs; 3, tissue; 4, broncho–alveolar aspirates; 5, wound pus; 6, endotracheal aspirates; and 7, sputum) and susceptibility to 10 antibiotics. (**a**) gentamicin, (**b**) amikacin, (**c**) ciprofloxacin, (**d**) ceftriaxone, (**e**) colistin, (**f**) fosfomycin, (**g**) ceftazidime, (**h**) imipenem, (**i**) meropenem, and (**j**) sulbactam.

The association between susceptibility test type and type of clinical isolate is illustrated for each antibiotic using a so-called ‘spaghetti plot’ (Fig. 5). Notably, most antibiotics had overlapping concentration lines for each type of clinical isolate in the MIC test, while MBEC50 and 75 discriminated between each type of isolate. The concentration of four antibiotics (colistin, fosfomycin, ceftazidime, and ciprofloxacin) was skewed heavily towards MBEC75 (more than two-fold) for isolates from urine, nasal swabs, and broncho–alveolar aspirates. Meanwhile, isolates from nasal swabs displayed significant variation between MIC and MBEC50 for sulbactam and fosfomycin (*p* < 0.001) (Fig. 5). The variation between MBEC50 and 75 was less pronounced for isolates from wound pus: amikacin, ceftriaxone, and ceftazidime; tissue: ceftazidime and sulbactam; endotracheal aspirates: ceftazidime and fosfomycin; urine: sulbactam; and endotracheal aspirates: fosfomycin. Imipenem, and meropenem demonstrated a substantially similar pattern of variation (MBEC75 > MBEC50 > MIC) for all types of clinical isolates (Fig. 5).

### Relationship between sample type and biofilm formation capacity is clarified by the anti-biofilm test method

**Figure 6:**
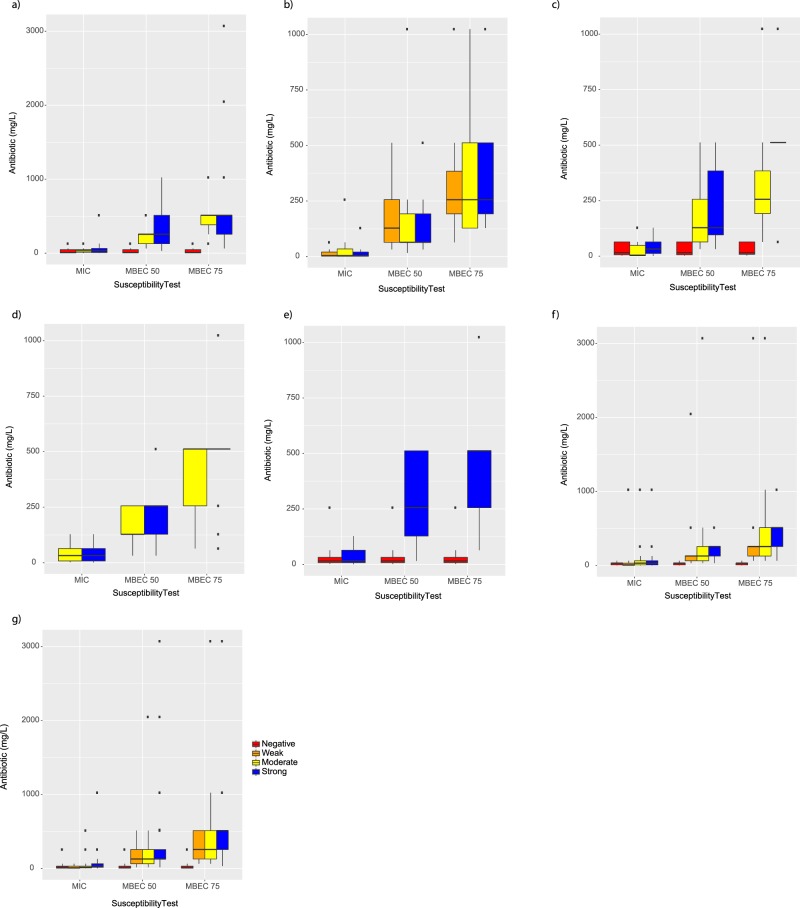
Relationship between susceptibility test results, biofilm formation (negative, weak, moderate, or strong) and type of clinical sample (1, urine; 2, nasal swab; 3, tissue; 4-broncho–alveolar aspirate; 5, wound pus; 6, endotracheal aspirates; and 7, sputum).

In Fig. 6 we plot a systematic comparison of biofilm formation capacity with each type of clinical isolate to support the efficacy of susceptibility tests. We found that when the level of biofilm formation was incorporated, nearly all types of clinical isolates exhibit consistent variation with either MBEC50 or 75. The moderate and strong biofilms show reasonable similarity for MBEC50 in nasal swabs, broncho–alveolar aspirates, endotracheal aspirates, and sputum. The isolates from urine, tissue and broncho–alveolar aspirates predominantly formed strong and moderate biofilms, while only isolates from wound pus predominantly formed strong biofilms (Fig. 6).

### MBEC50 and 75 of antibiotics predict the capacity of isolates to form biofilms

We used standardized values of concentrations (*Z*-scores) to avoid higher (raw) values of concentrations that make the associations appear more trivial (Table 1). This analysis found that both MBEC50 and 75 tests have significant capacity to classify biofilm formation accurately (*p* < 0.001) for the antibiotics used compared with a MIC test. The MBEC50 and 75 data showed a high consistency to predict correctly the biofilm formation as “negative” or “weak” or “moderate” or “strong”. The MBEC75 tests clearly predicted biofilm formation more accurately than the MBEC50 test, and higher divergence was seen for imipenem, meropenem, and sulbactam.

**Table 1:** Accuracy of biofilm classification (“negative” or “weak” or “moderate” or “strong”) from ordinal mixed effect regression by susceptibility results for each of the antibiotics based on standardized (*Z*-score) concentrations.

Antibiotic	MIC^†^	MBEC50^‡^	MBEC75^‡^
Gentamicin	48.55%	65.22%	68.84%
Amikacin	54.35%	69.57%	68.84%
Ciprofloxacin	48.55%	64.49%	65.22%
Ceftriaxone	46.38%	52.17%	54.35%
Colistin	44.93%	52.17%	64.49%
Fosfomycin	44.93%	57.25%	64.49%
Ceftazidime	48.55%	60.87%	65.22%
Imipenem	57.24%	94.92%	95.65%
Meropenem	63.04%	86.95%	97.10%
Sulbactam	44.92%	74.63%	73.91%

^†^Minimal inhibitory concentrations (MIC, μg mL^−1^) for planktonic cells.

^‡^Minimal biofilm eradication concentrations (MBEC, μg mL^−1^) were categorized as responsive reaching about 50% and 75% of the total non-viable cells within a given antibiotic concentration range.

The percentage (%) represent prediction of biofilm classification (“negative” or “weak” or “moderate” or “strong”) accuracy for all tested antibiotics based on standardized (*Z*-score) concentrations.

## Discussion

Identifying a response of biofilms to antibiotics accurately using a simple reliable and standard assay has remained a major limitation in selecting adequate antibiotic therapy^19^. In the first part of our work, we altered the several technical parameters to optimise assay performance. For example, a ratio of 1:10 PrestoBlue reagent to cell culture volume resulted in a stronger fluorescent signal with a shorter incubation time, and a more accurate signal-detection linear range than other resazurin-based assays^21,25–29^. Furthermore, as twofold less staining reagent was used to compare with other approaches^21,25–29^, this substantially reduced possible sources of false positives because of reduced fluorescent signal noise, as well as false negatives, and minimized reagent expense.

Assays requiring high fluorescence specificity are particularly vulnerable to inappropriate incubation times and reading modes^30,31^. For *A. baumannii*, we have shown that similar fluorescent intensity can be obtained with a bottom and a top reading mode with a 30-min incubation time. Therefore, this approach produces a more accurate and reproducible assay, in which both reading modes could be used either sequentially or in an interleaved fashion. In the present assay, the fluorescence intensity changes over extended incubation times for both reading modes. Reasons for the changes include that resorufin photobleaches, in which the fluorescence decreases over time because of its exposure to excitatory light or is reduced to colourless non-fluorescent hydroresorufin over time^21^. Importantly, one study has assumed that rather than using an endpoint mode, addition of resazurin staining with the test drug can produce more stable fluorescent signals^25^. Our observations raised questions for such an assumption and the reproducibility of that procedure. The capacity of resazurin to generate a fluorescent signal sufficiently adequate above background usually lasts for up to 1–4 hours^30^. Therefore, early exposure to staining with a test drug only produces fluorescent signals during the first several hours and prolonged exposure to excitatory light can decrease the signal-to-background ratio and sensitivity^30^.

We noted a difference in fluorescent signals from bacteria in suspension and those from bacteria in biofilms, either because of differences in the metabolic activity of the bacteria within the biofilms, such as the low metabolic activity of persister cells^3^ or perhaps because of limited visibility or accessibility of fluorophores to bacteria within the biofilms, preventing or delaying some fluorescence signal as a result of the physical structure of the biofilm. We found that the calibration curves with planktonic bacteria may not be relevant to estimate CFUs in biofilms. Our staining approach showed wider linear correlation between the fluorescent signal and the biofilm CFU counts than reported in earlier studies. This correlation allowed detection of <10^4^ CFU for *A. baumannii* biofilms across all the antibiotics tested. The technical validation provides data to support that the assay is sufficiently sensitive to detect low numbers of cells (low CFU counts) for selecting a suitable MBEC^31^. Therefore, such specificity for biofilms leads to higher sensitivity and precision than conventional resazurin-based assays^21,25–29^.

When the performance of an anti-biofilm assay is applied to the treatment of Acinetobacter infections, there is a need to assess the assay performance and reliability with clinical isolates. Our results showed the proposed anti-biofilm assay (MBEC50 and 75) was able to achieve and provide a clear delineation of antibiotic efficacy between the various capacity of biofilm formation (weak, moderate, or strong). These results re-emphasize the sensitivity of the assay and ability to resemble clinical situations. As a result, understanding how these different biofilm formation responses to antibiotics afford the credibility for clinicians to drive accurate therapeutic strategies toward biofilm-associated infections. In particular our results, the relationship between biofilm formation capacity and susceptibility test results reflect that the character of bacterial cells within biofilms have a large impact on antibiotic efficacy. This emphasized that in addition to their propensity to evolve and acquire inherited resistance to survive in high concentrations of antibiotics^32^, bacteria that are inherently susceptible to tested antibiotics can also be phenotypically refractory to their action via biofilm-mediated tolerance^5^. Also, our results clearly emphasized the assumption made in previous studies that every clinical isolate produces a similar amount of biofilm is not true, and they have not clearly proven whether their assay was able to mimic diversity of biofilm formation. This also raises questions about the credibility of the biofilm formation assay reported in previous studies^17,19,20,22–24^.

Our MBEC results showed that at low concentrations (<MBEC50), the antimicrobials more commonly employed against Acinetobacter (ceftazidime, sulbactam, imipenem, and meropenem)^33^ do not entirely kill the bacterial biofilm of most of the tested isolates. This property can result in significantly poor efficacy of antibiotics for clinical isolates from urine, nasal swabs, tissue, broncho–alveolar aspirates, wound pus, endotracheal aspirates, and sputum. Our results show that isolates that are susceptible to β-lactams or carbapenems are distinctly less effective against eradicating biofilm. However, carbapenems have 57–83% clinical cure rates for *A. baumannii* ventilator-associated pneumonia (VAP) for an adequate period with frequent recurrence (despite possible clinical improvement)^34–36^. Planktonic cells (which cause an acute exacerbation) respond briskly to carbapenems, but are resistant via biofilm formation to establish persistent infection^34^. A rapid biofilm test (MBEC50 and 75) should facilitate confirmation of biofilm susceptibility to specific carbapenems before their use.

At present, evidence from MIC assays does not allow for any firm conclusions about the effectiveness of colistin on Acinetobacter biofilms. However, polymyxins, such as colistin, are the agents most commonly used for Acinetobacter isolates resistant to first-line agents^37^. By contrast, our MBEC test results clearly emphasized the effective concentration of colistin varies by biofilm formation capacity and sample type. Colistin can diffuse through biofilms and is able to achieve 75% non-viable cells with relatively lower dosage than other antibiotics tested. This observation suggests that relative effectiveness depends on penetration of antimicrobials through the biofilm matrix and different physiological activity of the bacteria in biofilms^2,4,38^.

Their restricted penetration through biofilms may result in exposure of bacteria to low concentrations of antibiotics for long periods^39^. This exposure may fuel the emergence and selection of antibiotic resistant mutants with a potential risk of systemic spread to other organs or nosocomial spread to patients^3,39^. Our MBEC test results showed that the capacity of antibiotics to diffuse through biofilm varied with the type of antibiotic and sample. For example, gentamicin, amikacin, ciprofloxacin, and fosfomycin were shown to penetrate moderate biofilms readily compared with their penetration of strong biofilms, which is highlighted by the accurate predictability of MBEC50 and 75 results for biofilm formation. These antibiotics are commonly used to treat chronic respiratory, urinary tract, sinus, and ear infections^40^, but often fail to resolve them^2,5,41^. In all isolates, substantially higher concentrations of antibiotics were needed to achieve 75% cell death (MBEC75) than for MIC. Moreover, MBEC50 results of antimicrobial activity on biofilm formation capacity with the various sample types and antibiotics revealed that ciprofloxacin, ceftazidime, gentamicin, amikacin, and fosfomycin are effective only against the (metabolically active) outer layers. Whereas colistin can kill cells in the inner layers of biofilm preferentially (low MBEC75), which indicates unparalleled penetration and provides opportunities to establish combination therapy (such as with ciprofloxacin or the β‐lactams).

Biofilm infections are associated with various human milieu^2,3,12^ that allow various structural characteristics and complex resistance spectra^2^. Therefore, an anti-biofilm assay should be effective where the biofilm formative capacity of related infections differs between the sites of infections, so the antibiotic selection for each site will be more reliable. For example, our MBEC results showed the samples from people with chronic lung infections or endotracheal tube infections may produce a different profile of responses to antibiotics and displayed significant variation compared with MIC results. This suggests current MIC antibiotic testing may not lead to appropriate antibiotic choices for infections that are associated with biofilm infections^2,5^ and result in recurrence of symptoms after treatment^2^.

Examining the anti-biofilm efficacy of antibiotics using our assay will increase the selection of effective therapy for chronic biofilm infections. First, the assay provides for rapid, simple, and accurate identification of anti-biofilm sensitivity patterns allowing the most potent and effective drug to be selected. The selection of an optimal drug may also contribute to minimize bacterial resistance and spread of further infection or recurrence. Selection of a specific antibiotic therapy can contribute to preserve healthy gut flora, supporting immunity and health. Second, having a detailed understanding of the anti-biofilm effectiveness based on sample type guides implementation of the therapy best suited to a particular infection site or type (e.g., local or systemic). Third, the accurate classification of biofilm formation from MBEC data demonstrates the utility of the anti-biofilm test. We consider that empirical antibiotic therapy for chronic Acinetobacter infections should be selected based on patterns of biofilm-specific susceptibility, and addition of a biofilm-specific susceptibility assay will facilitate appropriate treatment selections. We observed strong agreement across the clinical isolates that MBEC50 provides an important base line for predicting the efficacy of antibiotics for eradicating biofilms. A 50% reduction of bacterial viability within a biofilm may be beneficial for patients with chronic and recurrent pneumonia because of high Acinetobacter load because such a reduction would facilitate more immune cells to access the bacteria within the biofilm and contribute to bacterial clearance^2^. However, patients with an immunodeficiency disorder are unable to effectively resolve infections or other complications related to their immune system, such as peritoneal dialysis, and so MBEC75 may provide better antibiotic selections to control their infections, such as chronic and recurrent pneumonia. Therefore, integration of the anti-biofilm assay into clinical settings will aid the application of accurate and effective antimicrobial therapy.

The anti-biofilm approach presented here offers the potential of broad applicability to determine the efficacy of antibiotics through their effects on biofilms. Yet, appropriate standard reference values required to clear infections *in vivo* remain unclear. To obtain a clinical effect on planktonic bacteria, antibiotics must achieve a >4 log_10_ reduction to fulfil performance standards^12,19^. However, there is no such kind of standard requisite log reduction value that best indicates therapeutic efficacy for biofilm infections^12^. Standardization of such values would improve therapeutic outcomes, and we welcome efforts in this direction. The microenvironments of infection sites where biofilms grow may not replicate precisely the nutrient-rich media used *in vitro* under assay conditions. Nevertheless, both the EUCATS^42^ and CLSI^43^ criteria use nutrient-rich medium methods for standard MIC drug testing *in vitro*. The experiments presented here are limited in that they have focused only on clinical isolates of *A. baumannii* and the assay may need various modifications before it can be applied to other species of bacteria. Moreover, only 10 antibiotics in current clinical practice were examined. More extensive testing in a similar fashion with other antibiotics would strengthen the credibility of the present assay.

In conclusion, our assay may be advantageous for the treatment of chronic infections with *A. baumannii*, but clinical trials are required to confirm this assertion. The assay is a valid, simple, reliable, and yet robust testing platform on which to dissect the antibiotic sensitivity of biofilms of *A. baumannii*.

## Materials and Methods

### Bacterial strains and growth conditions

After approval of the study protocol by the Institutional Review Board of the Faculty of Medicine, Chulalongkorn University, Bangkok, Thailand (COA No. 745/2017, IRB No. 414/60), *A. baumannii* clinical isolates (n = 138) with various morphology and resistance profiles were obtained without preference from a strain repository at the Department of Microbiology, King Chulalongkorn Memorial Hospital. These strains were stored in a repository collection after standard characterization and identification, including 16S rRNA sequencing. Clinical strains used in this study had been isolated during 2016–2017 from 137 chronically infected patients and represented 7 collection sites (including urine, nasal swabs, tissue, broncho–alveolar aspirates, wound pus, endotracheal aspirates, and sputum) as part of the standard care of the patients and was unrelated to the present study. Strains from patients with multiple sites of infection were excluded, and samples from patients with infection at only single site were included. Those *A. baumannii* (ATCC 19606) biofilm-positive strain and clinical isolates were cultured on Müller–Hinton agar (Sigma-Aldrich) plates at 37 °C. The strains were stored at −80 °C in tryptic soy broth (Sigma-Aldrich) with 15% glycerol until they were used in subsequent experiments in which they were suitably anonymised.

### Antibiotics and agents

The biofilm eradication activity of ten antibiotics was tested against a subset of isolates (n = 137) with reference strain ATCC 19606. Gentamicin, amikacin, ciprofloxacin, ceftriaxone, colistin, ceftazidime, imipenem, meropenem, and sulbactam were purchased from Sigma-Aldrich. Susceptibility testing for fosfomycin (Wako Chemicals) was determined by supplementation with 25 μg/mL glucose-6-phosphate (Sigma-Aldrich). Antibiotic stock solutions were prepared less than 24 h before use. Antibiotics were dissolved in cation-adjusted Müller-Hinton II broth (MHIIB) (Becton Dickinson) medium and the supplemented medium sterilized by filtration through a membrane filter nominally with 0.22 μm pores. Serial dilutions of the antibiotic stocks were prepared in MHIIB medium immediately before use.

### Optimization of biofilm formation

*A. baumannii* (ATCC 19606) was used as a model organism to optimize parameters for biofilm formation in a 96-well-microtitre-plate format as described previously with modifications to make the procedure more compatible with routine laboratory practice^20^. Initially, a pure culture of a single colony of *A. baumannii* was inoculated into 2 mL of MHIIB medium in a tube and incubated in an orbital shaker (200 rpm) at 37 °C overnight for about 16 h. Subsequently, a subculture was prepared from the overnight culture by diluting it with fresh MHIIB medium to an optical density (OD) of 0.02 at 600 nm (5 × 10^7^ CFU mL^−^^1^) and 100 μL aliquots were added in triplicate to flat-bottomed 96-well polystyrene microtitre plates (SPL Life Sciences), with uninoculated MHIIB medium (100 μL) in triplicate as a negative control, the plates were incubated at 37 °C for 24 h. After standardizing the conditions, we used the procedure to test the 117 biofilm-positive, and 20 biofilm-negative clinical isolates for their antimicrobial susceptibility profile under biofilm growth conditions. All experiments were performed in triplicate and repeated three times.

### Optimizing fluorescence signal quality

The 96-well-microtitre plates were incubated in darkness at 37 °C for six different times (15, 20, 30, 60, 120, and 240 min). The fluorescence of the contents of each well was measured (excitation 535 nm and emission 590 nm) using two optional reading modes (top from above the plate and bottom from below the plate) using a microtitre-plate-reading fluorimeter (Varioskan Flash Multimode Reader; Thermo Fisher Scientific). MHIIB medium and a blank control were used to correct for the background signal from each well. The parameters signal window coefficient *Z*′-factor, signal-to-noise (S/N), and signal-to-background (S/B) were calculated using the corresponding formulae: S/N = (mean signal − mean background)/SD of background, S/B = mean signal/mean background, *Z* = 1 − ((3 SD of sample + 3 SD of control)/(mean of sample − mean of control). The relationships between the fluorescent signal generated by the reduced resazurin and bacterial concentrations in the wells for both (planktonic) suspensions and biofilms of bacteria were analysed. First, for (planktonic) bacterial suspensions, dilutions of an exponential phase bacterial culture (from 2.80 × 10^3^ to 2.80 × 10^8^ CFU mL^−^^1^) were prepared in 96-well microtitre plates. PrestoBlue (Invitrogen) was added directly to the wells (10 μL/well) and the plates were incubated in darkness at room temperature for 20 min and then the fluorescent signal measured as described above. Second, various biofilm concentrations were achieved by incubating suspensions (exponentially grown, 2.80 × 10^3^ CFU mL^−^^1^, 100 µL/well) in 96-well plates for various times ranging from 1 to 24 h. Biofilm formation was confirmed by Crystal Violet staining^40^, followed by confocal laser scanning microscopy using live or dead bacteria staining as described previously^15^. Before staining, any non-adherent cells were removed from the mature biofilms by three gentle washes with MHIIB medium, and PrestoBlue was added (10 μL/well) as described above. The mean fluorescence values for test strains and negative controls were determined in triplicate and assays were repeated three times. To measure actual bacterial concentrations for planktonic suspensions and biofilm, CFU counts were quantified using conventional plating techniques from replicate wells. The number of CFU per biofilm in each well represented the number of bacteria cells within the biofilm after biofilm formation. Before counting the CFU, any non-adherent cells were removed from the mature biofilms by three gentle washes with MHIIB medium and biofilms were scraped vigorously from the well surface, serially diluted in MHIIB medium, and plated on MHIIA.

### Testing susceptibility to antibiotics

The MIC were established using standard techniques according to criteria in the EUCAST (criteria for *Enterobacteriaceae* for fosfomycin only)^42^ and CLSI guidelines^43^. *E. coli* ATCC 25922, and *P. aeruginosa* ATCC 27853 were used as quality control strains, with modifications as follows. To establish planktonic MIC for the antibiotics used, the antibiotics were serially diluted two-fold in 96-well microtitre plates (from 0.015 to 4098 μg mL^−^^1^) and bacteria added. The plates were incubated at 37 °C for 18 h. Minimal biofilm eradication concentrations (MBEC) were established by adding the serially diluted antibiotics to mature biofilms and incubating at 37 °C for 24 h and then staining with PrestoBlue. Before adding the antibiotics, any non-adherent cells were removed from the mature biofilms by three gentle washes with MHIIB medium. Cell viability was calculated using the following formula: cell viability (%) = ((mean signal of corresponding well − mean signal of negative control well)/(mean signal of positive control well − mean signal of negative control well)) × 100. Two cut-off values (50% and 75% non-viable cells) were used to determine the MBEC. Pearson correlations of PrestoBlue reduction to CFU/mL was analysed using the R statistical package^44^. All experiments were performed in triplicate and repeated three times.

### Biofilm quantification and classification

Two methods were used to quantify^45^ and classify^46^ the biofilm by Crystal Violet staining with modifications. Crystal Violet (0.1%) stained biofilms were solubilized with 30% acetic acid followed by incubation at room temperature for 10–15 min. The absorbance (OD) at 550 nm was determined using a microtitre-plate-reading spectrophotometer with 30% acetic acid as a negative control. Mean absorbances and their standard deviations (SD) were calculated for all strains and negative controls tested, performed in triplicate and repeated three times. The cut-off value (OD_β_) was defined as 3 SD above the mean OD of the negative controls: OD_β_ = average OD of the negative controls + 3 SD of negative controls, and was calculated separately for each microtitre plate. The OD of a tested strain was expressed as the mean OD of the strain minus the OD_β_ (OD = mean OD of a strain − OD_β_). The clinical isolates were classified as described previously^46^.

### Statistical analyses

Variables are described using standard deviations and means for continuous variables, and counts and percentages for categorical variables. The levels of drug susceptibility were represented in two ways: a continuous measure of concentration; and an ordinal categorical form representing biofilm formation (negative, weak, moderate, or strong), and both of these outcomes were measured repeatedly over time for each isolate, we employed mixed modelling to analyze these longitudinal outcomes. Linear mixed modelling was used to compare concentrations (quantitative) between test types over time. We then examined which test better discriminated between biofilm formations (negative, weak, moderate, or strong) using ordinal logistic mixed effects regression. For both types of mixed models, we considered both random intercept and random coefficient models, using model parsimony (as represented by Akaike’s Information Criteria) to choose the better model. Finally, we examined whether concentration could be used to predict biofilm formation using multi-nominal logistic regression. This approach is not unlike running separate binary logistic regressions for each pair of outcome values, subsequently yielding corresponding odds ratios for each pair, but multinomial logistic regression has the added advantage of providing an omnibus significance test of the overall model. All analysis was conducted using the R statistical package^44^. The linear mixed modelling was performed using the R library, lme4^47^, ordinal logistic mixed effect modelling using the R library, ordinal^48^ and multi-nominal logistic regression using the R library, nnet^49^. A significance level of *p* < 0.05 was used throughout all inferential analysis.

### Ethics approval

The study protocol was approved (COA No. 745/2017, IRB No. 414/60) by the Institutional Review Board of the Faculty of Medicine, Chulalongkorn University, Bangkok, Thailand, and was performed in accordance with the ethical standards as laid down in the 1964 Declaration of Helsinki and its later amendments and comparable ethical standards.

### Informed consent

For this type of study of anonymised clinical isolates formal consent from patients was not required.

## Supplementary information

Supplementary Informations

## Data Availability

The data specific to the clinical isolates that support the findings of this study are available on upon reasonable request from the corresponding author TC. Because of privacy/ethical restrictions (COA No. 745/2017, IRB No. 414/60) by the Institutional Review Board of the Faculty of Medicine, Chulalongkorn University, Bangkok, Thailand, the data are not publicly available because clinical isolates were obtained from chronically infected patients as part of the standard care and information contained within them could compromise the privacy of patients.
