# Supplementary material for: Simple fluorometric-based assay of antibiotic effectiveness for Acinetobacter baumannii biofilms
Source: Sci Rep. 2019 Apr 19;9:6300. doi: 10.1038/s41598-019-42353-0 (PMC6474882; doi:10.1038/s41598-019-42353-0)

Simple fluorometric-based assay of antibiotic effectiveness for *Acinetobacter baumannii* biofilms

Dhammika Leshan Wannigama[1,2,9], Cameron Hurst[3,4], Lachlan Pearson[5,6], Thammakorn Saethang[5,11], Uthaibhorn Singkham-in[1,9], Sirirat Luk-in[1,7,10], Robin James Storer[8], Tanittha Chatsuwan[1,9, *]

[1]Department of Microbiology, Faculty of Medicine, Chulalongkorn University, King Chulalongkorn Memorial Hospital, Bangkok, Thailand

[2]Medical School, Faculty of Health and Medical Sciences, The University of Western Australia, Nedlands, Western Australia, Australia.

[3]Center of Excellence in Biostatistics, Faculty of Medicine, Chulalongkorn University, Bangkok, Thailand.

[4]Department of Statistics, QIMR Berghofer Medical Research Institute, Brisbane, Queensland, Australia.

[5]Systems Biology Center, Research Affairs, Faculty of Medicine, Chulalongkorn University, Bangkok, Thailand.

[6]Snowy River Vet Clinic and Veterinary Hospital, Orbost, Victoria, Australia

[7]Interdisciplinary Program of Medical Microbiology, Graduate School, Department of Microbiology, Faculty of Medicine, Chulalongkorn University, Bangkok, Thailand.

[8]Office of Research Affairs, Faculty of Medicine, Chulalongkorn University, Bangkok, Thailand.

[9]Antimicrobial Resistance and Stewardship Research Unit, Faculty of Medicine, Chulalongkorn University, Bangkok, Thailand.

[10] Department of Clinical Microbiology and Applied Technology, Faculty of Medical Technology, Mahidol University, Bangkok, Thailand.

[11] Department of Computer Science, Faculty of Science, Kasetsart University, Bangkok, Thailand.

Supplementary information

Supplementary Table 1.

Betas with 95% confidence interval (95% CIs) from linear mixed modelling by compare concentrations (quantitative) between susceptibility test types for each of the antibiotics over time.

Supplementary Figure 1. Correlation between the number of CFU per biofilm as determined by plating (CFU count) and biofilm cell viability as a percentage of *A. baumannii* ATCC 19606 exposed to increasing concentrations of antibiotics.

Supplementary Figure 2. Distribution of the PrestoBlue-stained viable cells (coloured bar chart) and CFU counts (coloured symbols) of antibiotic-treated biofilms of *P. aeruginosa* PA01.

Supplementary Figure 3. Distribution of biovolume and biomass within the biofilm in a 96-well plate in relation to initial bacterial concentration (CFU/mL) measured for planktonic bacteria and biofilms. (A) Biovolume data based on mean values of three independent replicates of z-stack measurements by confocal laser scanning microscopy, (B) Biomasses data based on

staining with crystal violet and mean values of three independent replicates of absorbance (OD) measurement at 550 nm.

Supplementary Figure 4. Dose–response curves of antibiotics towards biofilm and planktonic cells were generated measuring PrestoBlue cell viability as a percentage in an assay validation step in *A. baumannii* ATCC 19606.

Supplementary Figure 5. Quantification of biovolume distribution in clinical isolates of *A. baumannii* biofilm after washing step using different washing solutions. Biovolume data are mean values of three independent replicates z-stack measurements by confocal laser scanning microscopy.

Supplementary Table 1.

Antimicrobial agents	MIC†			MBEC-50‡			MBEC-75‡		
	95% CIs§		β‖	95% CIs§		β‖	95% CIs§		β‖
	L95	U95		L95	U95		L95	U95	
Gentamicin	-31.13618	72.81897	20.8414	96.11921	168.75035	132.4348	282.78588	355.41702	319.1014
Amikacin	-3.877788	104.7358	50.4290	145.527550	228.2985	186.9130	344.049289	426.8203	385.4348
Ciprofloxacin	-9.457032	27.81935	9.181159	74.293791	127.01056	100.652174	252.844516	305.56128	279.2029
Ceftriaxone	-6.432496	149.2263	71.39689	208.807024	289.3959	249.10145	369.270792	449.8596	409.56522
Colistin	-7.873465	16.84595	4.486242	27.226724	49.36748	38.297101	64.328174	86.46893	75.39855
Fosfomycin	42.45418	245.7491	144.1016	103.27316	344.6254	223.9493	347.84562	589.1979	468.5217
Ceftazidime	1.800552	96.66574	49.23314	122.490231	191.68368	157.08696	288.055448	357.24890	322.65217
Imipenem	-27.52334	101.6767	37.07669	273.24233	385.2794	329.26087	578.86551	690.9026	634.88406
Meropenem	-40.85145	95.59948	27.37401	285.77460	400.02250	342.89855	588.61518	702.86308	645.73913
Sulbactam	-32.55944	37.54344	2.491998	129.75117	183.27781	156.514493	264.70769	318.23434	291.471014

[†]Minimal inhibitory concentrations (MIC, mg mL^{-1}) of planktonic cells.
[‡]Minimal biofilm eradication concentrations (MBEC, mg mL^{-1}) were categorized as responsive reaching about 50% and 75% of the total non-viable cells within a given antibiotic concentration range.
[§] 95% confidence interval
[‖] Beta

Suplimentry Figure 1

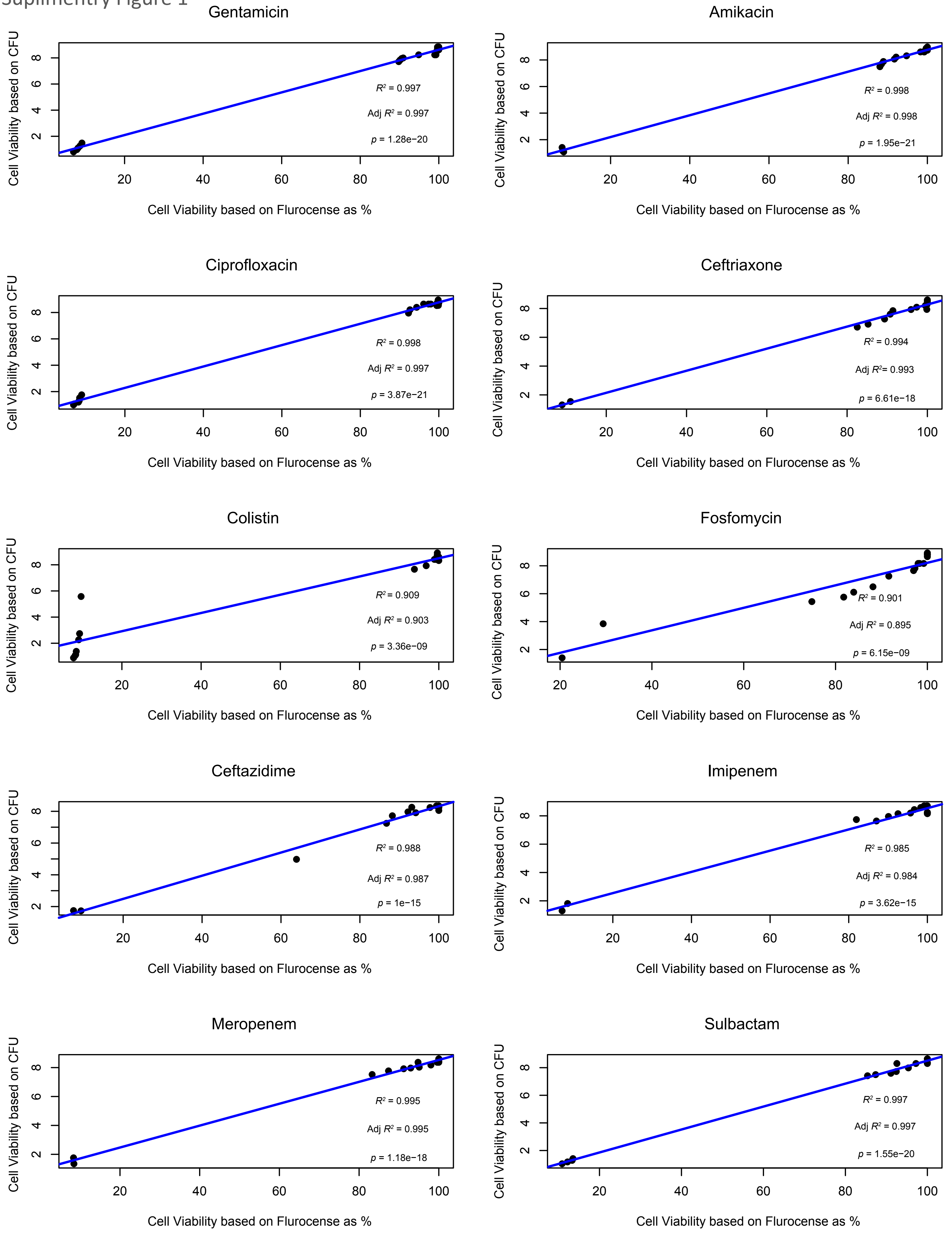

Supplementary Figure 2

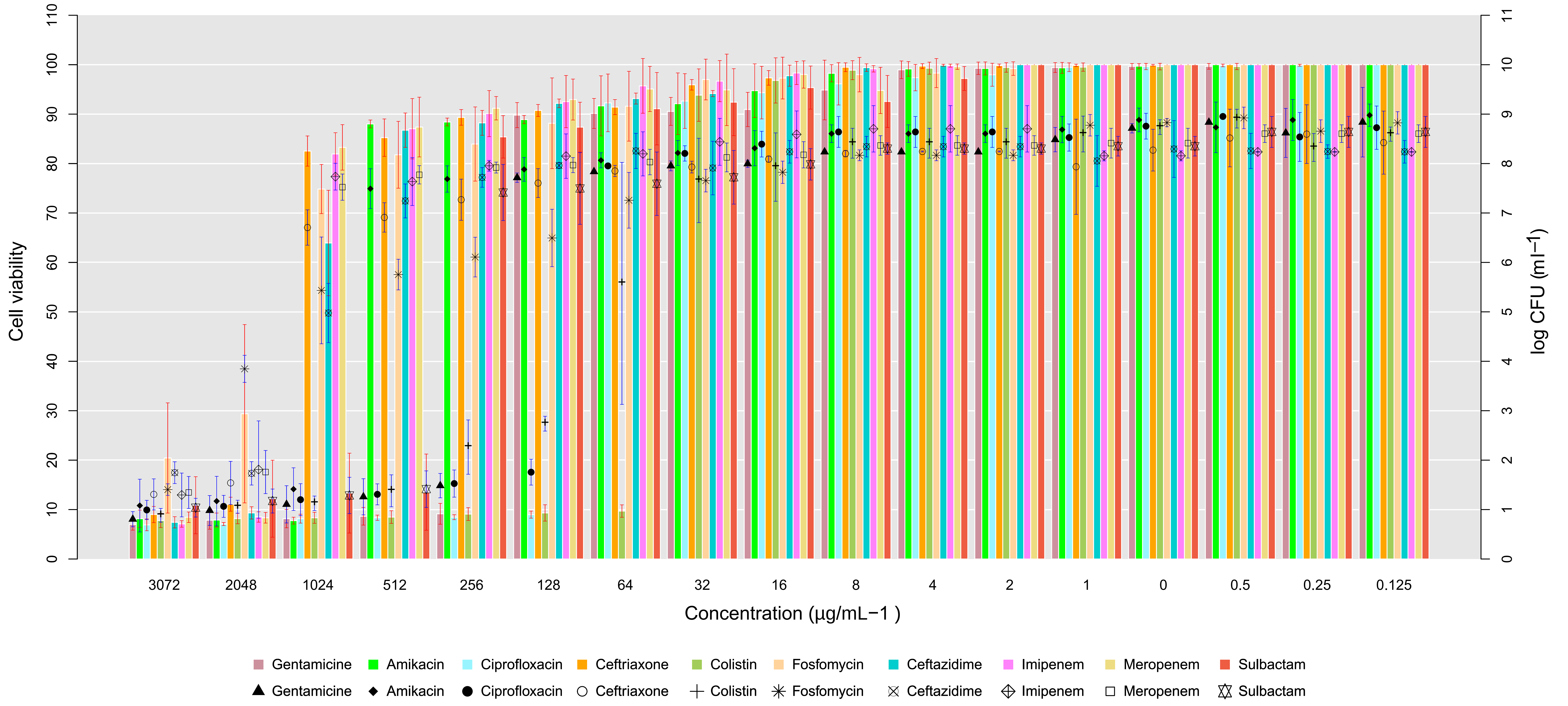

Supplementary Figure 3

a)

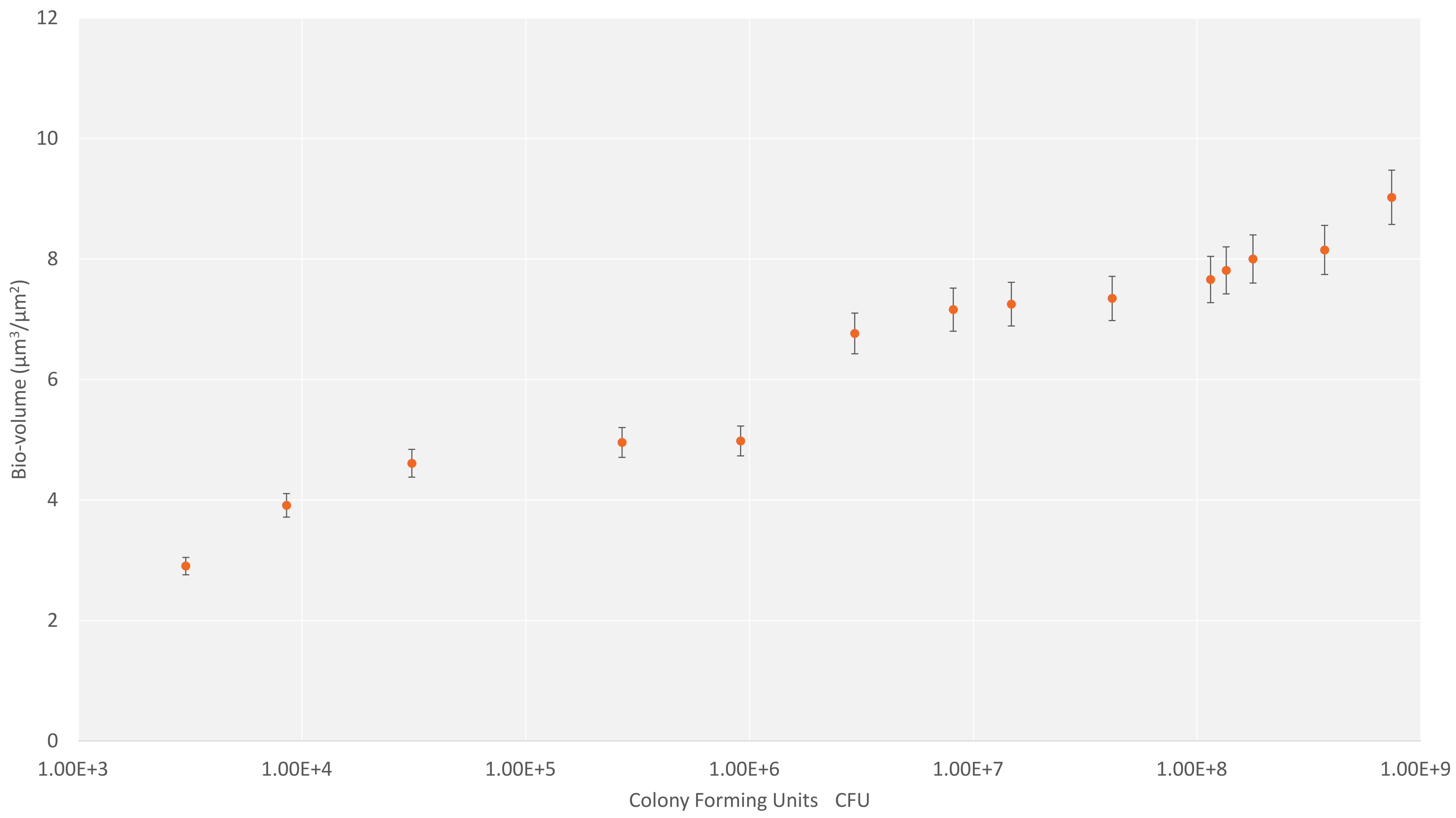

b)

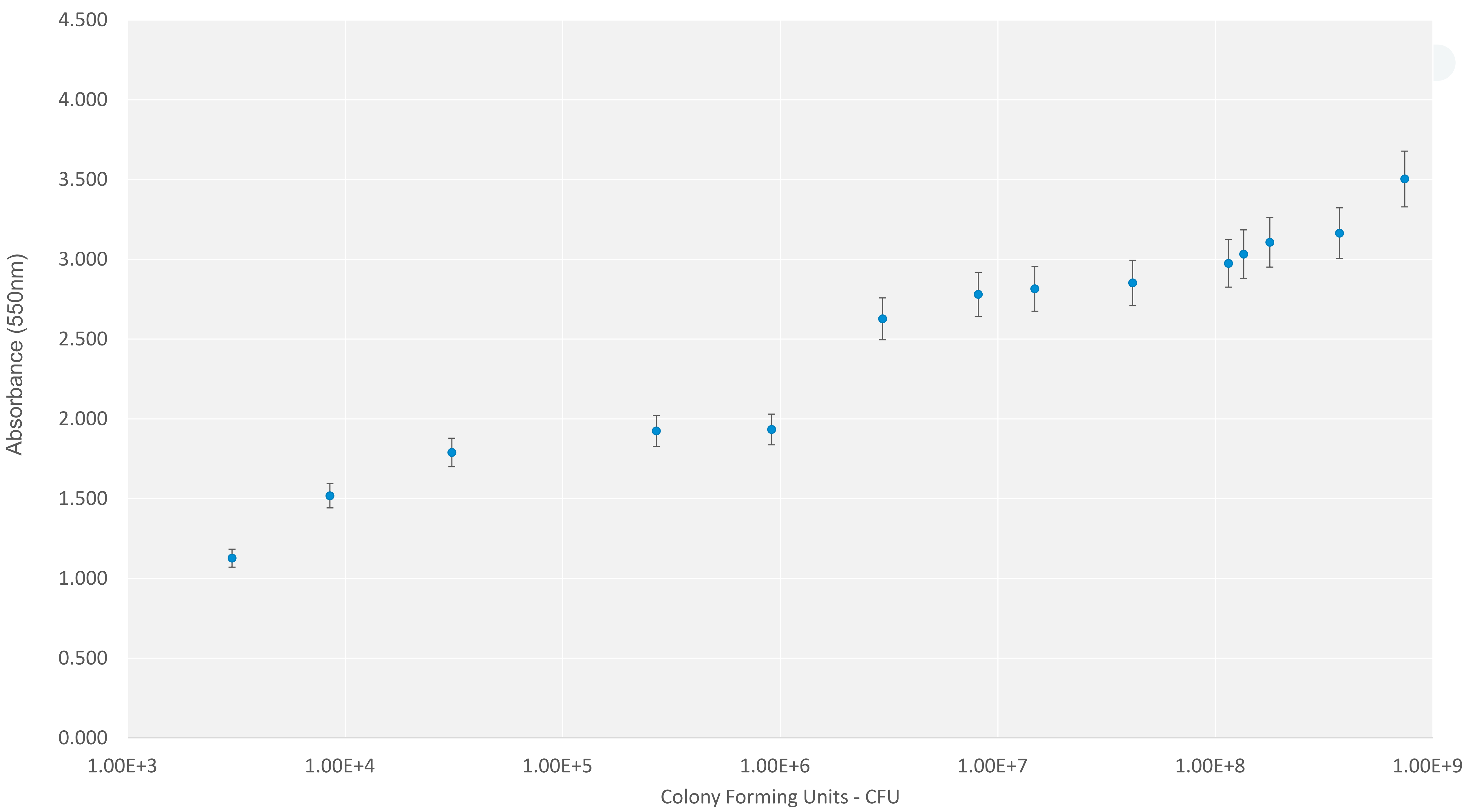

Supplementary Figure 4

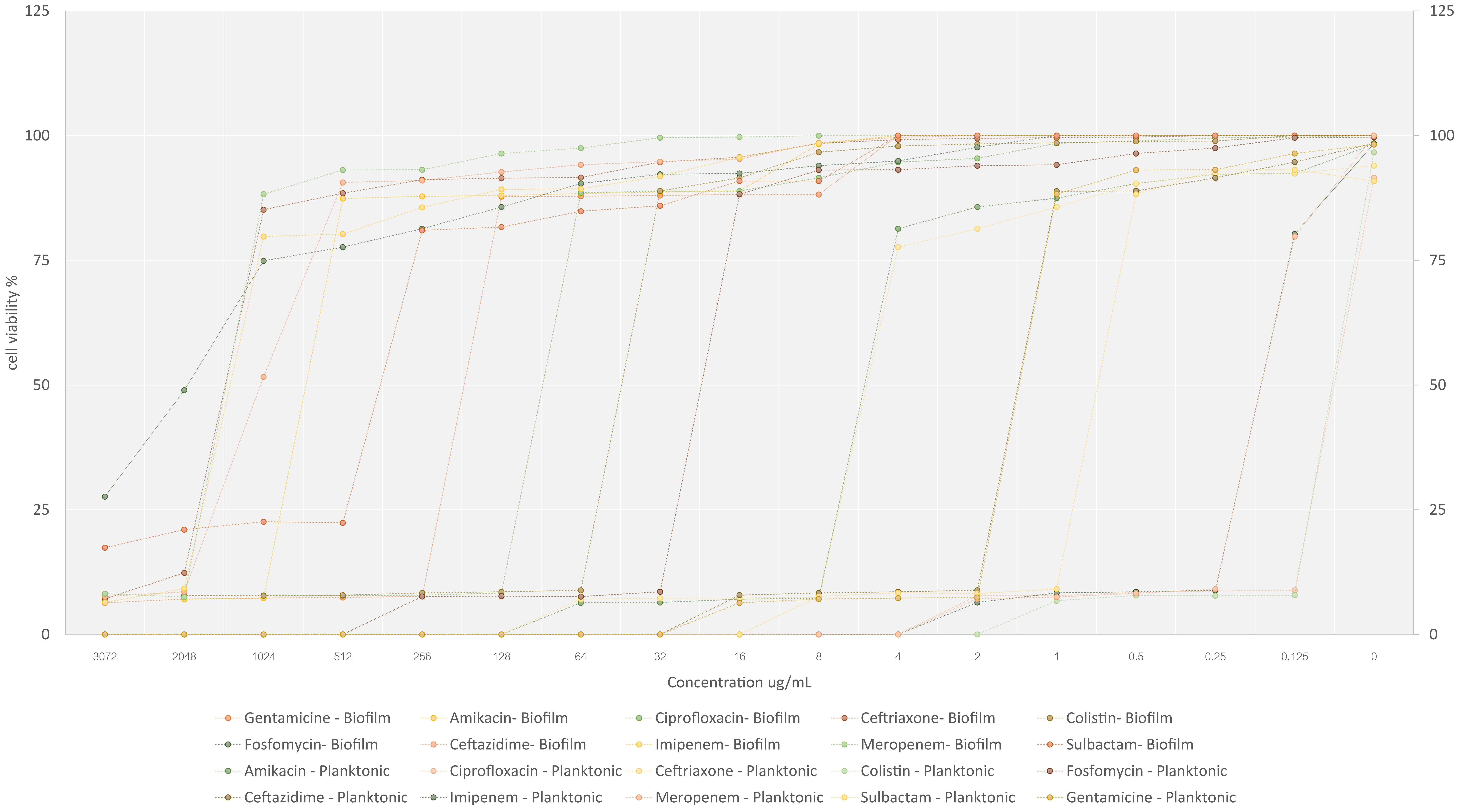

Supplementary Figure 5

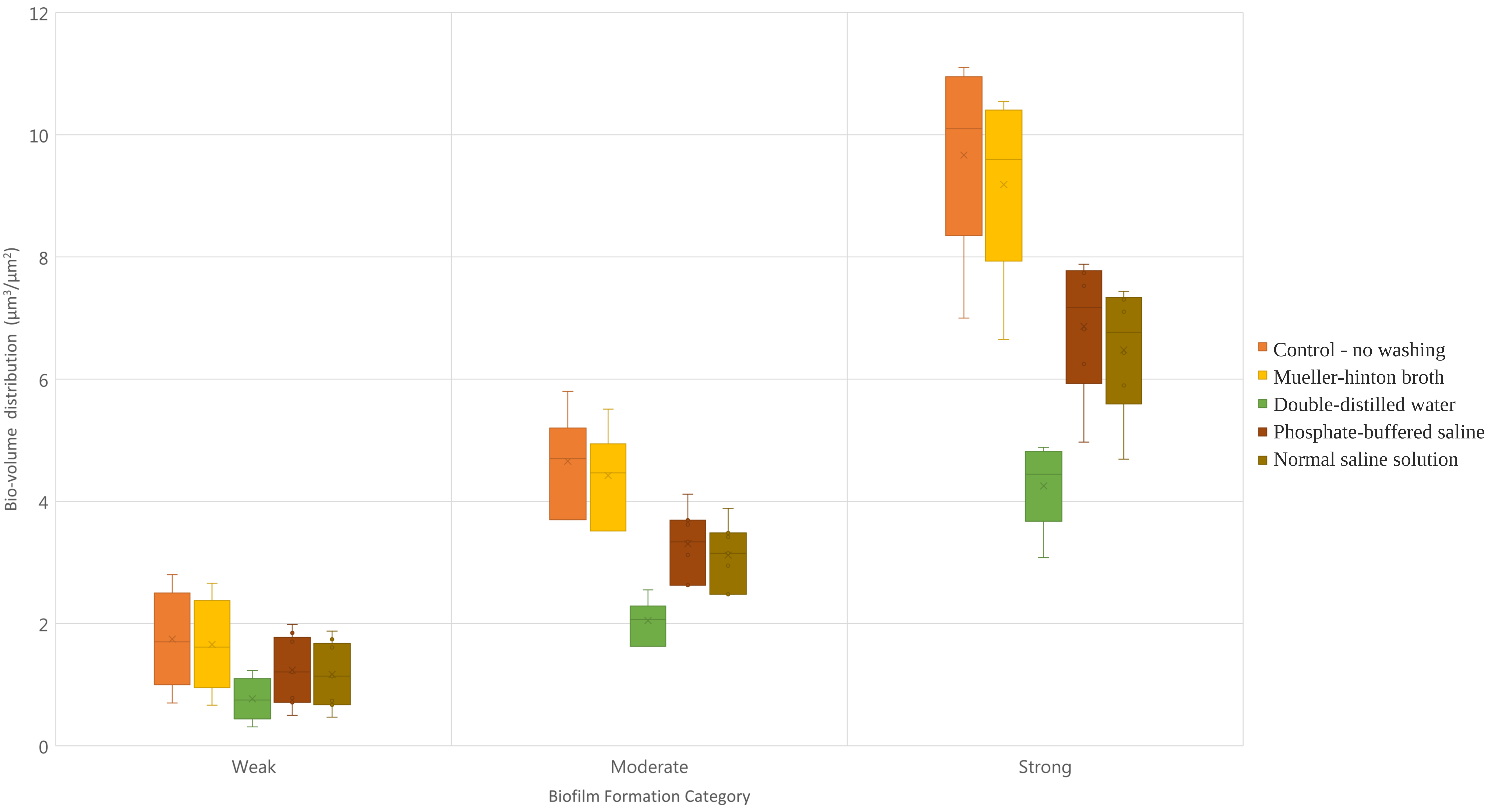

Supplement: Supplementary file 1 — Supplementary Informations [file 41598_2019_42353_MOESM1_ESM.pdf]
